# Cognitive Dysfunction after On-Pump Operations: Neuropsychological Characteristics and Optimal Core Battery of Tests

**DOI:** 10.1155/2014/302824

**Published:** 2014-04-30

**Authors:** Anna G. Polunina, Elena Z. Golukhova, Alla B. Guekht, Natalia P. Lefterova, Leo A. Bokeria

**Affiliations:** ^1^A. N. Bakulev Research Center for Cardiovascular Surgery, Russian Academy of Medical Sciences, Rublevskoe shosse 135, Moscow 121552, Russia; ^2^Moscow Research and Clinical Center for Neuropsychiatry of the Healthcare Department, Ul. Donskaya 43, Moscow 115419, Russia; ^3^Department of Neurology and Neurosurgery of Russian National Research Medical University, Leninsky pr-t 8–8, Moscow 119049, Russia

## Abstract

Postoperative cognitive dysfunction (POCD) is a mild form of perioperative ischemic brain injury, which emerges as memory decline, decreased attention, and decreased concentration during several months, or even years, after surgery. Here we present results of our three neuropsychological studies, which overall included 145 patients after on-pump operations. We found that the auditory memory span test (digit span) was more effective as a tool for registration of POCD, in comparison with the word-list learning and story-learning tests. Nonverbal memory or visuoconstruction tests were sensitive to POCD in patients after intraoperative opening of cardiac chambers with increased cerebral air embolism. Psychomotor speed tests (digit symbol, or TMT A) registered POCD, which was characteristic for elderly atherosclerotic patients. Finally, we observed that there were significant effects of the order of position of a test on the performance on this test. For example, the postoperative performance on the core tests (digit span and digit symbol) showed minimal impairment when either of these tests was administered at the beginning of testing. Overall, our data shows that the selection of tests, and the order of which these tests are administered, may considerably influence the results of studies of POCD.

## 1. Introduction

Postoperative cognitive dysfunction (POCD) is a mild form of perioperative ischemic brain injury, which emerges predominantly as memory decline during several months, or even years after surgery [1–5]. In addition, patients commonly report decreased concentration, attention span, and psychomotor speed. Both patients and spouses of patients notice postoperative memory decline after the first year after operation [3]. Negative effects on driving abilities during several months after uncomplicated cardiac surgeries were registered [6].

Some researchers include a broader spectrum of disorders around the term “postoperative cognitive dysfunction”—these disorders may include perioperative ischemic brain damage, brain death, delirium, stroke, and transitory ischemic attack [5]. Multiple neuropsychological studies confirm a decline in the performance of cognitive tests in the majority of patients during the first week after cardiac surgery and in about 30–40% of patients in 1–3 months after surgery in comparison with preoperative testing [review: [7]].

In young patients, cognitive function tends to be restored to its preoperative levels, in about 6–12 months after surgery [8–12], and there is no association between POCD and risk of dementia, in this young patient population, as was found in the study of Steinmetz and colleagues [13]. However, in some patients POCD may persist or even progress after 5 years of their operation, and the quality of life is significantly affected—this is directly related to the POCD level, on patient's long-term follow-up [14]. Moreover, Steinmetz and colleagues [13] found an increased mortality rate during a median of 8.5 year follow-up in patients with POCD, which was registered in 3 months after operation. In the same patient population, risk of leaving the labor market prematurely was twice as high among patients with 1-week POCD in comparison with patients without POCD.

Neuroimaging studies consistently demonstrate an acute brain edema and global decrease of brain metabolism as common, during the first three days after on-pump surgery [15–18]. In addition, 20–45% of on-pump patients demonstrated multiple small ischemic lesions postoperatively, and only 20% of them showed clinical signs of stroke or delirium [18–22]. Hence, mild brain ischemic injury is common after on-pump surgery. Overall, the total volume of brain ischemic alterations correlates with clinical symptoms in the majority of neuroimaging studies [21–24].

Significant associations between microembolic load, and severity of postoperative ischemic brain injury have been consistently found [16, 25–27]. Postoperative cognitive dysfunction was associated with intraoperative cerebral microembolic load—this was shown in a range of studies [27–29]. More importantly, Stygall and colleagues [30] found a significant association between cognitive decline, 5 years after CABG (coronary artery bypass grafting), and the number of intraoperative microemboli, in their study. Hence, cerebral microemboli appear to be a very important factor for brain ischemia in the majority of patients undergoing on-pump operations.

Cerebral hypoperfusion, during on-pump surgery, is also commonly addressed as an important factor contributing to postoperative brain damage. However, its significance appears to be especially prominent in atherosclerotic patients [31–33]. In addition, hypoperfusion impairs the clearance of microemboli from cerebral circulation, and as a result, hypoperfusion worsens ischemic damage, induced by microemboli [34–36]. The systemic inflammatory response is discussed as another important contributor to perioperative brain damage, as the concentration of inflammatory cytokines and C-reactive protein considerably increases after on-pump operations [37, 38].

It should be noted that problem of intraoperative cerebral microembolism, and POCD is not limited to cardiac surgery settings. Orthopedic surgery is associated with excessive microembolism as well [39–41]. Indeed, any vessel or skin trauma may lead to air or fat embolism, especially when a wound is elevated in comparison to the heart level and when multiple veins are open [42]: mini-invasive manipulations may induce massive microembolism [43, 44], and POCD is also commonly found, after noncardiac and off-pump surgeries [4, 5, 45].

Neuropsychological testing remains the most popular approach for the evaluation of mild postoperative neurological deficits. We analyzed 160 publications concerning neurological complications after cardiac surgery, which were published from 1998 to 2002 [46]. We found that 64% of studies used neuropsychological tests for assessment of neurological outcomes and 45.6% used this method as the only approach for evaluation of neurological outcomes of cardiac operations.

The evaluation of neurological outcomes of surgical interventions by perioperative psychometric testing is an intensively criticized approach. Experts point out the absence of a “gold standard” for effective application of this method in cardiac surgery studies [4, 5, 45, 47–51]. Interestingly, many findings in this field were not confirmed by other analogous studies, done by other research groups; therefore, they are considered to be highly arguable and unclear. However, it is an established fact that repeated neuropsychological evaluations lead to changes in performance, independent of any changes in underlying function or capacity, due to practice effect, or regression to the mean phenomenon [45, 50–52].

Moreover, several studies demonstrated a discrepancy between postoperative complaints in specific cognitive areas, and neuropsychological testing results [2, 53]; therefore, ecological validity of a range of tests is questionable. For instance, Vingerhoets and colleagues [2] did not find significant intergroup differences from preoperative test results on Rey auditory verbal learning test (RAVLT), following a 1-year follow-up between patients complaining of postoperative memory decline and patients not complaining—similar findings were characteristic for concentration, attention, and psychomotor speed domains in this study. At the same time, the group with cognitive complains had significantly higher anxiety and depression, as compared to the patients without complains.

The selection of tests remains problematic and varies extensively between studies. Newman and colleagues [4] have counted 70 different neuropsychological tests which have been used in the studies of postoperative cognitive dysfunction, published before December 2005. Comparisons between studies are difficult because of the differences in the tests selected. This variability in choices of tests may be due to the absence of a clear, theoretically derived and empirically tested model, which describes the causes and outcomes of postoperative cognitive changes [5].

Preliminary recommendations were given by the “Statement of Consensus on Assessment of Neurobehavioral Outcomes after Cardiac Surgery” in 1995 [54]. The authors suggested four neuropsychological tests (Rey auditory verbal learning test, trail making test parts A and B, and Grooved Pegboard test) as a core battery for research of POCD. This standard was used in many studies after 1995, and now the results of the neuropsychological studies in the cardiac surgery field may be compared and analyzed.

In our analysis of 24 publications concerning neuropsychological outcomes of cardiac surgery [55], we found that studies which used a test battery recommended by Statement of Consensus on Assessment of Neurobehavioral Outcomes after Cardiac Surgery (i.e., RAVLT, TMT A and B, and Pegboard test) showed significantly lower incidence of POCD after 1–3 months of follow-up, as compared to the studies which used digit span and nonverbal memory tests (average incidence: 28–32% versus 45–52%).

In addition, all (*n* = 13) studies which used Pegboard test, for assessment of POCD during first week after cardiac surgery found significant impairment of performance in the studied patient cohorts, whereas only 4 of 20 (5%) studies reported the same trend at their 1–3 month follow-up [7]—very similar dynamics was characteristic of TMT A and B, digit symbol, reaction time tasks, letter-cancellation, and Stroop test (i.e., neuropsychological probes with high loading on psychomotor speed processes). At the same time, postoperative deficits in tests with memory/learning components (RAVLT and other word list learning tests, digit span, nonverbal memory tests) showed less reversible postoperative deficit with significant decline of memory/learning function after 1–3 months follow-up, in 12–44% of studies.

It may be concluded that different types of POCD exist. Early psychomotor slowing is highly reversible and disappears after the first postoperative week in the majority of patients. Memory/learning deficit is more stable and may be detected during at least three postoperative months.

In our original study which using intraoperative transcranial doppler monitoring [56], we registered significant linear correlations between the intraoperative microembolic load on the left hemisphere and postoperative decline of performance on digit span forward, but we did not observe this on the “word-list learning” or “story learning” tests. In addition, we observed significant correlations between intraoperative microembolic loads on the right hemisphere and a decline on “nonverbal memory” tests. Therefore, it may be concluded that some memory test paradigms relate to neurological reality more closely, in comparison with other memory tests.

Here we present the results of three neuropsychological studies conducted in Bakulev's Scientific Center of Cardiovascular Surgery of Russian Academy of Medical Sciences. The primary aim of Study 1 was to evaluate the association between intraoperative microembolic load and POCD [27, 56]. During this study, we noticed that high proportion of patients dropped out from the study due to difficulties while performing 10 neuropsychological tests. The present analysis of the data was purposed to determine the optimal tests which may be used for the registration of POCD in cardiac surgery patients in order to shorten the neuropsychological evaluation and decrease the drop-out rate.

Study 2 (which was primary aimed to evaluate the perioperative dynamics of brain electric activity [57, 58]) confirmed the effectiveness of our 5-test battery as a tool for detecting different types of POCD, for example, left hemisphere deficit, right hemisphere deficit, and psychomotor slowing in patients after open-heart and coronary surgeries. Using 5-test battery allowed to decrease drop-out rate, and these data are presented here.

In Study 3, we searched if an ordinary position of a test in a test battery may influence the study results. This issue is especially important when the test battery is short, and the neuropsychological deficit is mild. Although, it is logical that a test position may influence the study results, the effect of this factor on the dynamics of the performance on the concrete tests was poorly studied, especially in Russian speaking populations.

## 2. Study 1

### 2.1. Materials and Methods

#### 2.1.1. Patient Selection

The present study protocols were reviewed and approved by the Academic Council of Bakulev's Cardiovascular Surgery Center on the March 13, 2002. The study design was explained to patients, and each patient gave an informed consent to participate. Exclusion criteria were a history of stroke or other neurologic or psychiatric disease, reoperative surgical procedures, and inability to perform the cognitive test battery due to visual problems or non-Russian-speaking. Inclusion criteria were age 16–69 years old, cardiac ejection fraction > 40%, and availability of MCAs (middle cerebral arteries) to be insonated through the transtemporal windows. Each patient completed the clinical interview, which included items concerning their previous medical treatments, visits to a psychiatrist, and current medication intake in order to determine if a patient fulfilled the criteria of the study.

Healthy subjects, which were invited to participate in the study as controls, were recruited from the spouses of the patients and medical personal of the clinic. Subjects were between the ages 16 and 69 years, with the absence of serious neuropsychiatric and somatic diseases.

#### 2.1.2. Anesthesia, CPB, and Surgical Management

All patients were operated on by the same surgeon (L.B.) and the same team at Bakulev's Cardiovascular Surgery Center. The protocols of anesthesia and surgical techniques were standardized. Diazepam and morphine served as premedication. Anesthesia was induced and maintained with propofol, fentanyl, and pancuronium. The perfusion apparatus consisted of the Stökert S3 roller pump (Germany), DIDECO-703 membrane oxygenator (Italy), and a 40 *μ*m arterial filter. Nonpulsatile pump flow rates were maintained between 2.4 and 2.6 L∗min⁡^−1^∗m^−2^, and the mean perfusion pressure was at 60 mm Hg. The operations were accomplished during moderate hypothermia (28°C). All patients underwent median sternotomy, the aorta was cross-clamped, and the heart was arrested with anterograde cold pharmacological cardioplegia by solution of custodioli. Topical ice saline was used as an adjuvant to myocardial protection. During CABG, proximal anastomoses were made after removal of aortic cross-clamp using a partial occlusion clamp.

#### 2.1.3. Neuropsychological Assessment

Patients completed eleven neuropsychological tests in 2-3 days prior to surgery and then completed 10 of these tests in 2–4 weeks postoperatively. Testing was carried out in an isolated room by a neurologist experienced in neuropsychological assessment (A.P.). The following cognitive domains were assessed:

*Auditory Memory Span*. (1) Digit span forward of Wechsler adult intelligence scale (WAIS) required subjects to repeat a series of digits that have been orally presented to them in forward order; the maximal span of numbers was included into the analysis; (2) digit span backward of WAIS required to repeat a series of digits in reverse order; the maximal span of numbers was analyzed.

*Word or Story Learning*. (3) Luria memory test included learning of 10 words during 5 trials—the mean number of words during 5 trials was included into the study; (4) logical memory test of Wechsler memory scale (WMS) required subjects to repeat as many semantic items in the short story as possible—the number of the repeated items was analyzed. 

*Nonverbal Memory*. (5) Benton visual retention test: the subjects were asked to reproduce from memory 10 designs after 10 seconds of exposure, one at a time, as exact as possible; the numbers of exact completed designs were included into the analysis. 

*Visual Reconstruction*. (6) The block design test of WAIS involved subjects to reconstruct the geometric designs using either four or nine, two-tone colored blocks; the raw score of the test was analyzed. 

*Psychomotor Speed*. (7) Digit symbol of WAIS involved subjects to code, within 90 seconds, as many digits into symbols as possible—raw score was analyzed; (8) trail making test part A (TMT A): the subjects were asked to connect 25 numbers consecutively, as quickly as possible—the time taken to complete the test was analyzed. 

*Executive Functions*. (9) Trail making test part B (TMT B) required subjects to connect numbers and letters in sequential and alternative order (1, A, 2, B, etc.)—the time of the performance was analyzed. 

*Global Cognitive Status*. (10) Minimental state examination (MMSE) test, included 30 simple questions and tasks in a number of areas (orientation in time and place, repeating and recalling list of words, arithmetic, language use and comprehension, non-verbal memory). The summarized score was analyzed.

*Intelligence*. (11) The information subtest of Wechsler adult intelligence scale (WAIS) required subjects to answer standard questions concerning general knowledge. The information subtest was only given preoperatively, and the raw score was included in the analysis.

Parallel forms of Luria memory test and Benton test were used to minimize learning effects. As the logical memory test included two stories, we used one story for the primary assessment and another story for the follow-up assessment. The administration of parallel forms of the memory tests was not counterbalanced, (i.e., all the patients performed the test forms in the same order). The parallel forms for other tests were not available. The performance on WAIS subtests and MMSE was scored by a common procedure [59, 60]. The performance on TMT was evaluated by the time needed to complete the tasks [61].

#### 2.1.4. Statistical Analysis

All analyses were performed using SPSS software for windows (SPSS 17.0, Chicago, IL, USA). The preoperative group characteristics were compared by Pearson *χ* ^2^-tests and ANOVAs, with Bonferroni corrections, where appropriate. Changes of cognitive performance at follow-up, in comparison to baseline assessments, were evaluated using repeated measures ANCOVAs, with diagnosis and sex as grouping factors and age as a covariate. The Mann-Whitney test was used for post hoc analysis of intergroup differences, as deltas of several tests were not normally distributed. The factor analysis of neuropsychological variables was conducted using principle component method, with varimax rotation. Spearman's method was used for correlation analysis of relationships between age and neuropsychological variables.

### 2.2. Results

#### 2.2.1. Group Characteristics

Ninety patients were included into the study and completed the baseline assessment. However, only 65 patients (29 CABG and 36 open-heart patients) completed postoperative testing, and 50 patients (21 CABG and 29 OH) and 12 controls completed all tests. Twenty-five patients dropped out due to delay of surgery (7 patients), refusal to complete tests at postoperative period (15 patients), postoperative brachial plexopathy (2 patients), and multiorgan insufficiency and consequent death (1 patient). Seven patients demonstrated transitory mild psychotic symptoms (visual hallucinations and disorientation) during first postoperative day, with further complete normalization of consciousness.

The baseline characteristics of the patient and control groups are presented in Table 1. The patients, after open heart operations, were younger in comparison with CABG and control group. All patients after CABG were males; therefore, gender distribution was different between the groups. Overall, healthy controls showed a better baseline cognitive performance, in comparison with healthy controls in the majority of probes. However, this trend reached significance, only with the Benton visual retention test analysis, and was almost significant in logical memory test. Also to note—as the age and sex distribution was different in all three groups, all ANCOVAs were completed with sex as the second fixed factor and age as a covariate.

#### 2.2.2. Changes of Performance on Neuropsychological Tests at Follow-Up

**Figure 1:**
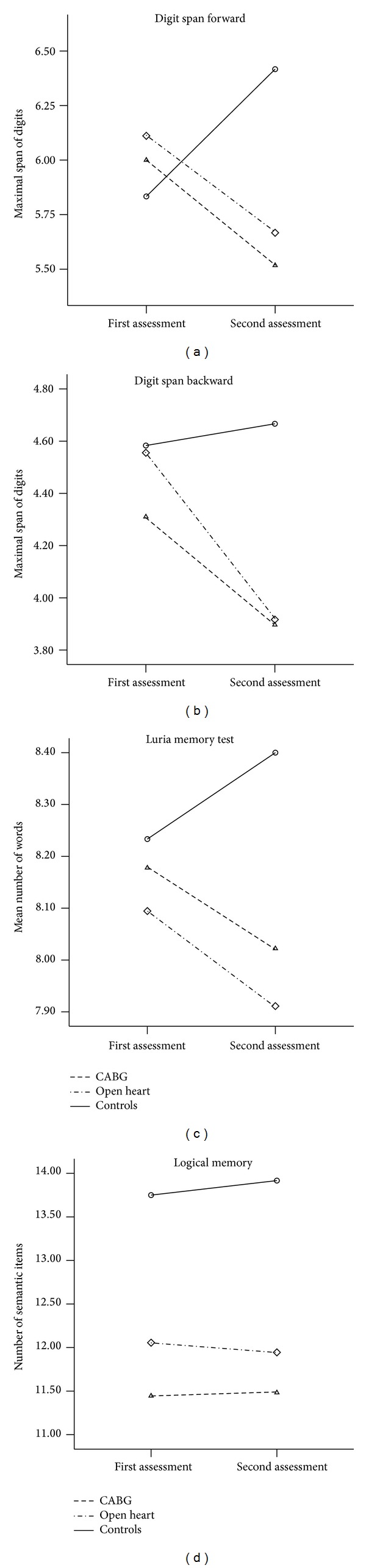
Changes of performance on auditory/verbal memory tests in three groups (Study 1; statistical results are presented in Table 2).

The results of the statistical analysis of intergroup differences of changes of performance, on 10 tests at follow-up in comparison to the baseline performance are summarized in Table 2. Both patient groups showed significant decline on verbal memory span (digit span forward and backward), whereas controls showed nonsignificant improvement in this cognitive domain at follow-up (Figure 1). Only the similar trend was registered for verbal learning (Luria memory test) and no intergroup differences were found for story learning (logical memory) test.

**Figure 2:**
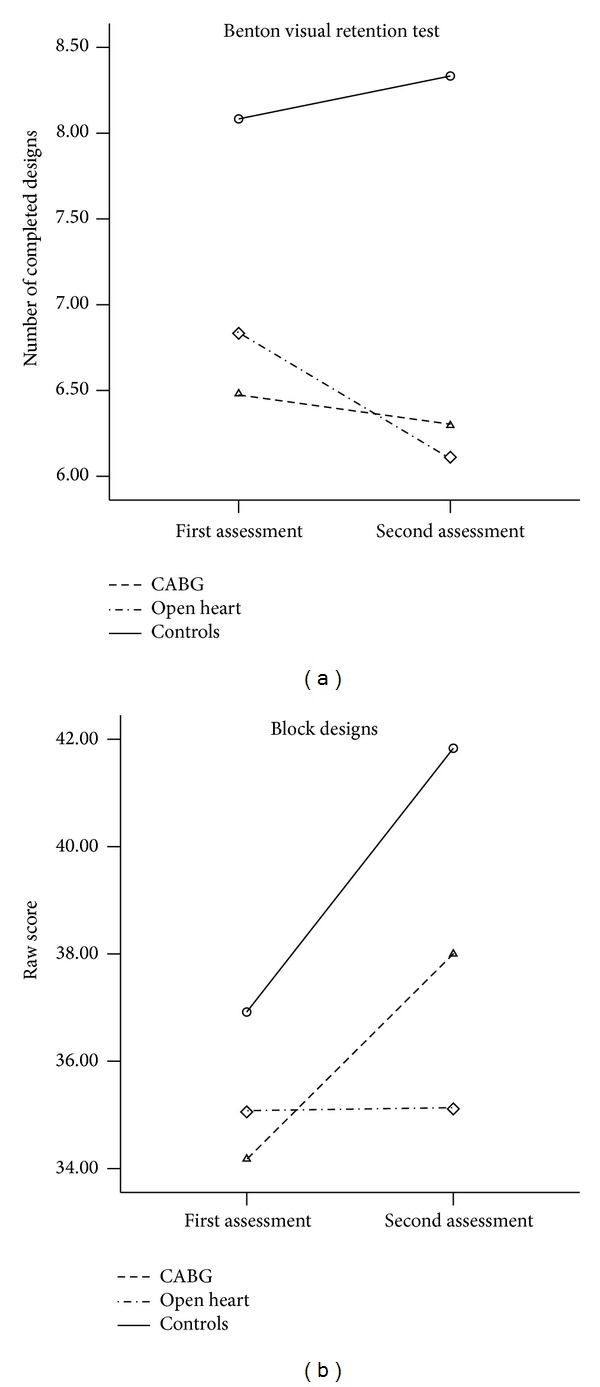
Changes of performance on nonverbal memory and visuoconstruction tests in three groups at follow-up (Study 1; statistical results are presented in Table 2).

Patients after open heart operations differed significantly from CABG and control groups by deficit of performance on non-verbal memory, and visuoconstruction tests at the postoperative period, in comparison with primary assessment (Figure 2). Dynamics on these neuropsychological parameters was similar in CABG patients and controls.

**Figure 3:**
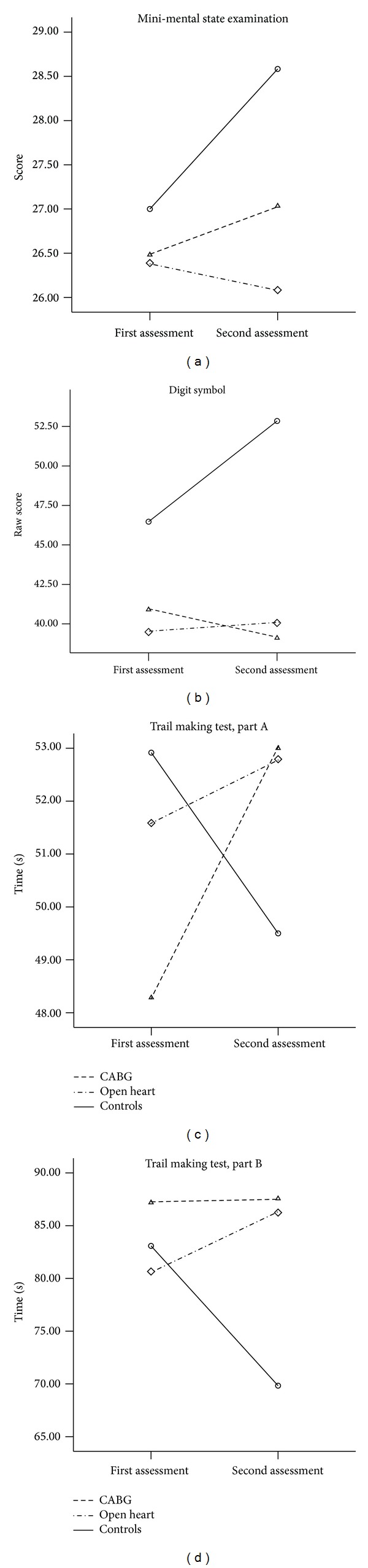
Changes of global cognitive status and psychomotor speed in three groups at follow-up (Study 1; statistical results are presented in Table 2).

Healthy subjects showed prominent practice effect on MMSE, whereas OH patients worsened their performance with intermediate results of CABG patients (Figure 3). In addition, healthy subjects completed timed tests (digit symbol, TMT A and B) more quickly at follow-up, in comparison with baseline assessment, whereas both patient groups either slowed or did not change their performance in this cognitive domain. Interestingly, CABG patients demonstrated significantly more pronounced slowing of psychomotor speed (digit symbol and TMT A), as compared to OH patients, in the postoperative period.

#### 2.2.3. Factor Analysis of Changes of Performance on Nonverbal Test

We suggest that changes at follow-up on different non-verbal tests may reflect changes in the same cognitive domains. In order to investigate this hypothesis, we conducted a factor analysis of deltas on nonverbal neuropsychological variables (Table 3). Three factors, which overall explained 63.4% of the variance of dynamics on the tests, were extracted. The Benton test and the MMSE contained nonverbal memory items, loaded on the first factor (22.2%). Two timed tests (digit symbol and TMT A) loaded on the second factor (19.7%), and the third timed test (TMT B) showed small but significant correlation with this factor. Most interestingly, TMT B and the block design test loaded on the same factor and therefore TMT B measured the same function as block design did in the context of our study.

#### 2.2.4. Age and Psychomotor Speed

Although repeated measures ANCOVAs included age as covariate, patients after CABG demonstrated decreased psychomotor speed after surgery, in comparison with OH patients. We suggest that an older age is an important factor influencing psychomotor speed slowing at postoperative period. Correlation analysis shows that age significantly correlated with performance on three timed test at preoperative (TMT A and B: *R*_s_ = 0.30 and 0.31, *P*_s_ < 0.01, resp.) and postoperative (digit symbol and TMT A: *R*_s_ = −0.24 and 0.37, *P*_s_ < 0.05, resp.) assessments. Perioperative dynamics of psychomotor speed significantly correlated with age as well (digit symbol and TMT A: *R*_s_ = −0.24 and 0.23, *P*_s_ < 0.05, resp.).

In addition, age significantly correlated with the baseline performance on the digit span backward, and logical memory test (*R*_*s*_ = −0.24and − 0.31, *P*_*s*_ < 0.05, resp.), with the similar trend for Luria memory test (*R* = −0.20, *P* = 0.074). At the second assessment, correlation between age and logical memory test was still significant (*R* = −0.35, *P* = 0.002), with a similar trend for the digit span backward and Luria memory test (*R*_*s*_ = −0.20 and − 0.21, *P*_*s*_ = 0.075).

#### 2.2.5. Effects of Sex on Cognitive Performance

Performance on the block design test was significantly higher in males compared to females, on both assessments (*F* = 7.83, *P* = 0.007). In addition, time * sex interaction was significant for the performance on the block design test; that is, females showed a larger improvement compared to males, on the test during the second assessment due to lower baseline performance.

Sex did not show significant effects on the baseline performance on the logical memory test; nevertheless, males showed slight improvement of the results during the second assessment—females demonstrated a slight decline (time ∗ sex interaction: *F* = 4.15, *P* = 0.045).

#### 2.2.6. Postoperative Delirium and Cognitive Dysfunction

Although few patients (*n* = 7) demonstrated mild psychotic symptoms during the first postoperative day, this subgroup was characterized by lower cognitive performance in comparison with patients without postoperative delirium at both preoperative and postoperative assessments. Patients with delirium showed significantly lower baseline performance on the MMSE (23.8 ± 3.7 versus 26.5 ± 2.5), digit symbol, logical memory, Benton visual retention test, and Luria memory test (*t*_*s*_ > 2.35, *P*_*s*_ < 0.05). At the follow-up, patients with delirium were characterized by significantly lower performance on the digit span forward and backward, MMSE, digit Symbol, Benton visual retention test, and Luria memory test (*t*_*s*_ > 2.02, *P*_*s*_ < 0.05). Performance on the digit span backward and MMSE decreased more prominently in patients with delirium in comparison with patients without delirium *t*_s_ > 2.05, *P*_s_ < 0.05.

### 2.3. Discussion

The present study showed three types of POCD in our patient sample: (1) both patient groups demonstrated similar postoperative decline on verbal memory tests; (2) patients after valve operations showed additional deficit in nonverbal memory and visuoconstruction tests; (3) elderly atherosclerotic patients were characterized by prominent postoperative slowing of psychomotor speed. Importantly, different tests appeared to measure deficit in the same neurocognitive domain. Some tests which traditionally are considered to relate to the same domain demonstrated different sensitivity to postoperative cognitive dysfunction.

We used four tests measuring verbal/auditory memory function, which is neurologically localized in the left hemisphere structures. We found that tests measuring auditory span were more sensitive to postoperative cognitive deficit, compared to word, or story learning. Moreover, in the same cohort of OH patients, we found a strong correlation between postoperative changes of the performance on digit span forward and intraoperative microembolic load on the left middle cerebral artery, whereas the correlation between postoperative changes of word list learning and microemboli only showed the same trend [56].

In CABG patients, we observed significant correlation between the length of cardiopulmonary bypass and postoperative changes of performance on the digit span forward, but not on word-learning or story-learning tests. Interestingly, our analysis of literature data [55] showed that studies which used digit span demonstrated significantly higher POCD incidence, compared to studies which did not use digit span (mean POCD incidence: 45.7 ± 23.3% versus 26.2 ± 18.0%, *t* = 2.63, *P* = 0.013). At the same time, studies which used RAVLT (word list learning) reported lower POCD incidence, compared to studies which did not use RAVLT (mean POCD incidence: 32.8 ± 21.0% versus 47.7 ± 25.1%, *t* = 1.81, *P* = 0.08). Overall, digit span appears to represent neurological reality more efficiently in the context of the postoperative cognitive dysfunction, when compared to word-learning or story-learning test paradigms.

Open Heart patients were characterized by a decline of visuospatial functions, in the present study. In the previous study [56] of the same OH patient cohort, we registered significant linear correlations between microembolic loads on the right middle cerebral artery and postoperative decline of the performance on Benton visual retention test (*R* = −0.39, *P* = 0.018); and overall microembolic load and postoperative deficit, in the block design test (*R* = −0.31, *P* = 0.04). It should be noted that both normal controls and CABG patients showed significant practice effect on the block design test at follow-up, whereas a deficit in OH patients was registered as an absence of practice effect, compared to two other groups. Overall, the OH group was characterized by almost twice as large a microembolic load, at the right MCA, compared to the CABG group; therefore, a deficit of right hemisphere functions was characterized by a larger microembolic load to this brain structure, in OH patients.

Changes of the TMT B score tended to correlate with the microembolic load at the right middle cerebral artery in OH group as well (*R* = 0.28, *P* = 0.09). Moreover, postoperative changes of TMT B score loaded on the same factor as postoperative changes of Block Design score, that is, the dynamics of performance on two tests was related to the same cognitive domain in the context of cardiac surgery outcomes. Although TMT B is traditionally considered to be a test sensitive to prefrontal cortex injury, it is not a “pure” executive function test—it measures a series of cognitive abilities [62, 63]. In the present study, TMT B measured at least two cognitive domains, that is, visual search/orientation and psychomotor speed.

Both patient groups showed significant slowing of psychomotor speed at the postoperative assessment; however, this phenomenon was most prominent in CABG patients. Correlation analysis confirmed a significant association between an older age and the slowing of psychomotor speed at the baseline and postoperative follow-up—this function was especially sensitive to the effects of “on-pump” in elderly patients.

Overall, the battery of 10 tests was difficult to perform for patients who were both pre- and postoperative. Therefore, we had chosen only 5 tests for the second study. We preferred to include digit span for the assessment of the volume of short-term/verbal memory, due to its larger sensitivity to postoperative neurological outcomes, compared to the word list learning and story-learning. We included block design as a tool for assessment of visuospatial functions, because patients less commonly complained that this test was very difficult to perform in comparison to the Benton visual retention test.

Digit Symbol was chosen as a measure of psychomotor speed, as this test is more popular in Russia, and may be easier replicated in future Russian studies, compared to the TMT. Finally, we included MMSE, as it proved to be an effective tool for measuring postoperative changes of global cognitive status. Overall, we found the same three types of postoperative cognitive dysfunction in the second study. Importantly, the drop out rate among cardiac surgery patients was considerably lower in the Study 2 in comparison with the Study 1.

## 3. Study 2

### 3.1. Materials and Methods

#### 3.1.1. Patient Selection

The present study protocols were reviewed and approved by the academic council of Bakulev's Cardiovascular Surgery Center, on February 21, 2007. The study design was explained to patients, and each patient gave an informed consent to participate. Exclusion criteria were a history of stroke or other neurologic or psychiatric disease, reoperative surgical procedures, and inability to perform the cognitive test battery due to visual problems or non-Russian speaking. Inclusion criteria were ages 16–69 years old; cardiac ejection fraction > 40. Anesthesia, CPB, and surgical technologies were the same as in Study 1.

The healthy control group was partially the same as in Study 1 (*n* = 19). Additional 11 subjects, either spouses of the patients or medical personal, were included into the present study as controls. Ages 16–69 years, with the absence of serious neuropsychiatric and somatic diseases, was the inclusion criteria.

#### 3.1.2. Design of the Study

Patients underwent clinical, EEG, and neuropsychological evaluation, 2-3 days before surgery. The postoperative assessment was performed between 10–15 days after surgery. Controls were reevaluated within a 2-week interval as well. EEG data were published elsewhere [57, 58].

#### 3.1.3. Neuropsychological Assessment

Five neuropsychological tests were used in the present study: digit span forward, digit span backward, block design test, digit symbol test, and minimental state examination. The testing procedure was the same as in Study 1, but the testing was conducted by another doctor (N.L.).

#### 3.1.4. Statistical Analysis

All analyses were performed using SPSS software for windows (SPSS 17.0, Chicago, IL, USA). The preoperative group characteristics were compared by Pearson *χ* ^2^-tests and ANOVAs, with Bonferroni correction, where appropriate. Changes of cognitive performance at follow-up in comparison to the baseline assessment were evaluated using repeated measures ANCOVAs with diagnosis and sex as grouping factors and age as a covariate. The Mann-Whitney test was used for post hoc analysis of intergroup differences as deltas of several tests were not normally distributed.

### 3.2. Results

#### 3.2.1. Group Characteristics

Fifty two patients were included into the study, and 50 subjects completed tests at preoperative and postoperative assessments (two other patients refused the postoperative testing). Thirty-four patients underwent open-heart operations, and 16 patients constituted the CABG group. Four open-heart patients demonstrated transitory mild psychotic symptoms (visual hallucinations and disorientation) during the first postoperative day, with further improvement of neurologic status and complete normalization of consciousness, later on. The patient and control groups' characteristics are summarized in Table 5.

Gender distribution was different among three groups; as only one of the CABG patients was female, whereas about half of the patients in the OH and control groups were female. In addition, the GABG patients were significantly older than the open-heart patients and showed significantly lower performance in the MMSE and digit symbol, in comparison with the other two groups.

#### 3.2.2. Changes of Performance on Neuropsychological Tests at Follow-Up

**Figure 4:**
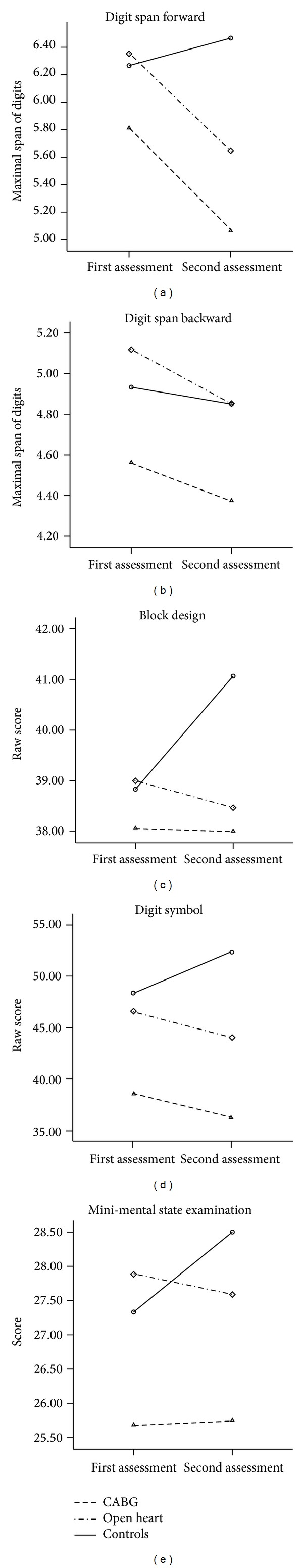
Changes of performance on neuropsychological tests in three groups at follow-up (Study 2; statistical results are presented in Table 4).

The results of neurocognitive testing of patients at the postoperative follow-up (Figure 4, Table 4) very closely resembled findings of Study 1: both patient groups were characterized by a significant decline on the digit span forward test. Overall, patients, after open-heart surgeries tended to show a more extensive neuropsychological deficit (though not significantly), compared to CABG patients, who had a more frequent involvement of visuospatial functions. Both groups demonstrated significant decrease of psychomotor speed at postoperative period as compared to the controls.

#### 3.2.3. Effects of Age and Sex on Cognitive Performance

Age was significantly associated with the performance on the digit span forward and backward, block design, and digit symbol tests (*F*_*s*_ = 12.0–24.2, *P*_*s*_ ≤ 0.001) at repeated measures analysis and significantly and negatively correlated with the performance on all tests, including MMSE, at both assessments (*R*_*s*_ = −0.26–0.42, *P*_*s*_ < 0.05). Sex was significantly associated with the performance on the digit symbol test due to better performance in females at both assessments (*F* = 6.50, *P* = 0.013).

### 3.3. Discussion

Study 2 confirmed the findings of Study 1 with a similar postoperative decline of auditory memory span and a psychomotor decrease in both groups; also, more extensive neuropsychological deficit involving visuospatial functions in open-heart patients was noted. Hence, the battery of tests including digit span, block design, digit symbol, and MMSE appeared to be quite sensitive to the three types of POCD (i.e., verbal/short-term memory deficit, visuospatial functions deficit, and psychomotor slowing), which we registered in the first study.

It should be noted, that the delay between pre- and postoperative testing was somewhat shorter in Study 2 in comparison the Study 1 (10–15 days versus 2–4 week). And this difference may underlie the poorer performance of open-heart patients on the digit symbol test in Study 2 in comparison with the open-heart patients in Study 1, despite of the similar young age.

## 4. Study 3

The purpose of Study 3 was to determine if an ordinal position of a test in a test battery may influence the study results.

### 4.1. Materials and Methods

#### 4.1.1. Patients

Study 3 was conducted in parallel to Study 1, with the same inclusion and exclusion criteria. Patients who refused to undergo intraoperative transcranial dopplerography or whose MCAs were unavailable for insonation through the transtemporal windows or due to the fact that other technical reasons were not available for transcranial dopplerography were invited to participate in this neuropsychological study. Overall, 30 patients (15 CABG and 15 OH) completed tests at preoperative and postoperative assessments. Demographic, clinical, and intraoperative characteristics of the present patient cohort did not differ from the larger patient population of Study 1 (see Table 1).

#### 4.1.2. Design of the Study

In order to investigate the effects of a test ordinal position in a test battery on the results of a study, patients were divided into three subgroups—each subgroup included 10 patients. The first subgroup* (Span-Symb-TMT)* completed a battery of tests in the following order: (1) digit span forward, (2) digit span backward, (3) Benton visual retention test, (4) Luria memory test, (5) digit symbol, (6) logical memory, (7) block design, (8) TMT A, and (9) TMT B. The second subgroup* (Symb-TMT-Span) *completed, in this order, digit symbol, TMT A, and TMT B at the 4th and 5th positions and digit span, at the end of testing. The third subgroup of patients completed the tests in the following order: TMT, Digit Span at the 5th and 6th position, and Digit Symbol at the end of testing. The tests and testing procedures were the same as in Study 1. Intergroup differences in postoperative performance, (in accordance with a test position) were analyzed.

#### 4.1.3. Statistical Analysis

All analyses were performed using SPSS software for windows (SPSS 17.0, Chicago, IL, USA). Intergroup differences according to the order of tests or diagnosis were evaluated using repeated measures ANOVA procedures, with an order of tests as a grouping factor. As perseverative errors of WCST were not normally distributed, the groups which performed neuropsychological tests only at the post-operative or preoperative periods were compared by Mann-Whitney tests.

### 4.2. Results

**Figure 5:**
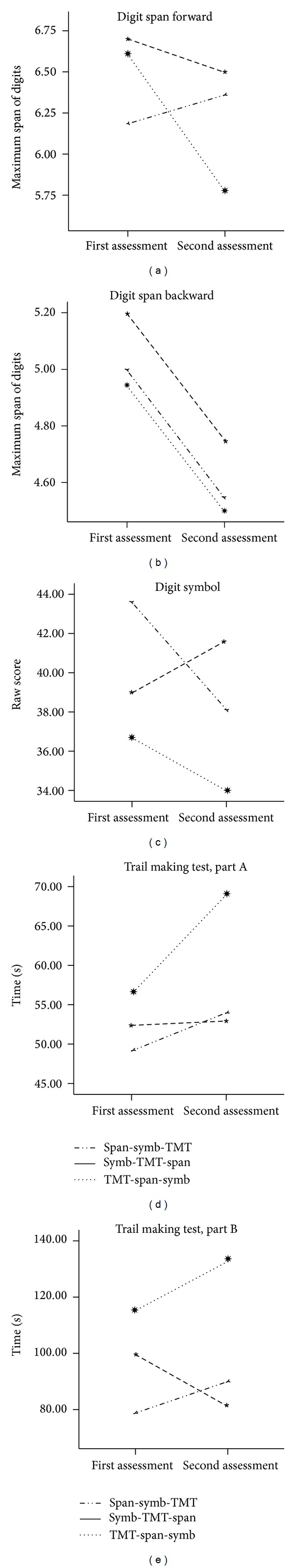
Effects of a test ordinal position on results of a study: the first position of the digit span forward (*F* = 4.03, *P* = 0.03), digit symbol, or TMT B in the battery of tests showed significantly different results from trials with the position of a test in the middle or end of the battery.

The distribution of CABG versus OH patients or males versus females among three groups did not differ (3-4 CABG patients; 5–7 males in each group). The age and education were similar as well. An ordinal position of a test in a test battery significantly affected results of the study in the present patient cohort (Figure 5). Patients which performed digit span forward as the first test in the battery showed significantly better dynamics of performance on this test at the postoperative period, compared to the patients who performed this test in the middle or end of testing (*F*_2,27_ = 4.03, *P* = 0.03). Patients who performed digit symbol as the first test in the battery showed significantly better dynamics on this test, compared to the patients who performed this test in the middle or end of the testing (*F*_2,27_ = 5.61, *P* = 0.01). No significant intergroup differences were found in the analysis of the postoperative changes of time, on TMT part A, or part B.

### 4.3. Discussion

To summarize, our data shows that different types of POCD exist and the neuropsychological tests and administration procedures used may considerably influence the results of a study. The postoperative decline of verbal memory span appears to be a universal type of POCD, which we found in both open-heart and CABG patient groups. In addition, postoperative involvement of visuospatial functions and psychomotor slowing were observed in specific subgroups of patients.

#### 4.3.1. Left Hemisphere Dysfunction

Several previous studies showed left hemisphere structures to be more prone to postoperative impairment, compared to the right hemisphere. Weinstein [64] found 75% of postoperative strokes to be localized in left hemisphere. Lee and colleagues [29] observed a decrease of blood flow in the left temporal region, after on-pump CABG, along with decrease of performance on word list learning test, without any changes in the right temporal region. Rasmussen and colleagues [65] found a decreased quantity of benzodiazepine receptors in the left temporal region, in on-pump CABG patients, three months after operation. Interestingly, strokes related to coronary angiography were predominantly localized in the left hemisphere, according to the study of Leker et al. [66].

The higher preponderance of cerebral infarcts in the left hemisphere was also reported in the general population of stroke patients [67–69]. This trend was shown to be more prominent in the strokes related to the middle cerebral arteries circulation [68, 69] and in young males versus young females [68]. In the older subjects, left hemisphere strokes were shown to be higher in severity and mortality, as compared to right hemisphere strokes [69]. The authors hypothesized that left hemisphere preponderance of ischemic strokes may be attributed to differences in the intima-media complex and slow velocity in the left carotid artery, resulting in higher stress and intimal damage, with earlier development of atherosclerosis, compared to the right hemisphere circulation [68, 69]. In addition, greater metabolic demands in the left hemisphere neuronetworks, compared with right hemisphere ones, may predispose left hemisphere structures to greater risk of functional decrement, after ischemic events [69].

Atochin and colleagues [70] showed that when microemboli were injected into a carotid artery to experimental animals, the former accumulated in ipsilateral, and even contralateral (through Willis circulation) temporal regions, whereas anterior and posterior circulations were less affected. Hence, it is logical that tests sensitive to functions of temporal lobe structures, that is, memory tests, are the most effective tools for POCD diagnostics.

Our findings that the digit span paradigm is higher in efficiency, compared to tests measuring short-term memory by word list learning or story learning approaches are consistent with two other studies, which also showed a higher correlation between digit span and cerebral microembolic load, compared to word list learning tests [28, 71]. It should be noted that we did not assess long-term memory (delayed recall, recognition) in our patient cohort, and further studies are needed for comparison between digit span and long-term memory assessment as tools for evaluation of POCD. Our preliminary experience concerning long-term memory assessments in cardiac surgery patients showed that the procedure of digit span demands less time and effort, compared to long-term memory assessments—and this circumstance appears to be important in the context of studying postoperative cognitive dysfunction.

Another limitation of our studies was the lower baseline cognitive performance in patient groups, compared to controls. Indeed, it is a common problem in the research of cognitive functions in medical patients, who commonly show lower cognitive performance in comparison with healthy populations. Nevertheless, according to the statistical phenomenon of “regression to the mean” [52], normal subjects with the lower performance at the first assessment would increase their results at the follow-up, whereas subjects with higher performance would decrease their results at repeated assessment, showing the trend to reach group means. Here, we observed further decrease of cognitive functions in patients, and this trend was opposite to controls, who showed the prominent practice effect at repeated testing.

#### 4.3.2. Right Hemisphere Dysfunction

We observed more extensive neuropsychological deficit involving visuospatial functions in patients after open-heart operations in comparison with CABG, in two different patient cohorts. There are a few previous studies which compared open-heart and CABG patients. One of them [72] found significantly higher incidence of deficit on the digit symbol test, in patients after valve replacement, compared to the CABG group (26.7% versus 6.8%, resp.), in the 6-month follow-up. Interestingly, Neville et al. [10] did not find intergroup difference, despite differing carotid embolic counts. Overall, neurological complications were consistently shown to be more common, after open-heart procedures, compared with on-pump CABG procedures [73–75].

In Study 1, we observed significantly higher right hemisphere embolism, compared to left one, in OH and CABG groups. However, there were, on average, twice as many emboli in OH patients, compared to the CABG group.

In open-heart patients, a decreased performance on the Benton visual retention test and an absence of the practice effect on the block design test significantly correlated with the number of emboli, which were registered on the right middle cerebral artery or sum of microemboli at both arteries, respectively. In the study of Borger and colleagues [71], a similar significant linear relationship between the performance on nonverbal memory tests and intraoperative cerebral microembolism was observed.

Although some cardiosurgical centers reported preponderance of the left hemisphere impairment in patients after cardiac operations [29, 64–66], other research groups found cardiac surgery related strokes [76], or decreased cerebral perfusion, to be more prevalent in the right hemisphere [77]. Moreover, an asymmetric distribution of microembolic load, during an on-pump operation, is diverse in different centers with preponderance of microemboli on the left hemisphere in one study [29] and predominantly right hemisphere microembolic load in another study [16]. These discrepancies mean that variations in surgical and perfusion technologies may considerably influence the distribution of intraoperative microemboli within the brain circulation system.

We used two tests sensitive to visuospatial functions in our series of studies, and both tests, that is, Benton visual retention test and block design test, were capable of registering postoperative decrement of accuracy of visuospatial processing in OH patients. Nevertheless, both patient groups showed significantly lower performance on the Benton test before surgery, compared to the controls, and commonly complained that the test procedure was excessively difficult to perform. Therefore, we decided to abandon the Benton test from our test battery for Study 2. At the same time, block design does not seem to be an ideal tool for diagnosis of right hemisphere deficit in patients after cardiac surgery, as this probe demonstrated a prominent practice effect in controls—an “impairment” on block design in OH patients was just an absence of practice effect, rather than true impairment. As a result, we suggest that a nonverbal memory span test would be a better choice for future studies of postoperative cognitive dysfunction.

#### 4.3.3. Psychomotor Slowing

Both patient groups showed postoperative slowing of performance on timed tests (i.e., digit symbol and TMT); however, this trend was especially prominent in atherosclerotic elderly patients in the CABG group. No correlation between microembolic load and postoperative psychomotor slowing was observed in our previous study [56]. This means that the mechanism of postoperative psychomotor slowing is different from the pathophysiology of memory dysfunction.

Psychomotor slowing is a typical neuropsychological consequence of the aging process [78], and deficit in this cognitive domain is especially prominent in atherosclerotic patients [79, 80]. Interestingly, longitudinal studies show that a proportion of patients, after cardiac surgeries, developed a “secondary” psychomotor slowing, with a normalization of this cognitive domain, several weeks after surgery, and worsening of it, after the 6-month or 3-year follow-up [28, 81]. The authors explained these findings as a result of the aging process, rather than looking at the effects of cardiac surgery. Overall, psychomotor slowing appears to be related to intraoperative microembolism, but not as directly as postoperative memory deficit does.

#### 4.3.4. Effects of a Test Position in a Battery on Study Results

We could not find previous studies on the effects of a test position in a battery on a study results. Therefore, our study is evidence that this is an important factor and should be taken into account. We observed that postoperative deficit on the digit span backward did not vary in three groups, even when this test was in the second position, in a test battery. Hence, it may be concluded that only the first position in a test battery may be of significance for study results.

## 5. Conclusion

The present study results give evidence that verbal memory span tests (digit span forward and backward) are more effective, compared to word-learning and story-learning tests as a tool for registration of postoperative cognitive dysfunction. Nonverbal memory span tests may be recommended for future studies as a tool for evaluation of right hemisphere functions. Finally, a psychomotor speed test (e.g., digit symbol) should be included into test batteries, as a tool for evaluation of POCD, which is characteristic for early postoperative period and aging process. According to the present results, these core tests should follow some other tests when administrated to cardiac surgery patients, which are less sensitive to the position in a test battery (e.g., TMT A and B), as the first position of a test in a battery may considerably influence the results of a study.

## Figures and Tables

**Table 1:** Patient characteristics (Study 1).

Variables	CABG	Open heart surgery	Healthy controls	*P *
Number of patients	29	36	12	
Male sex (%)	100*	52.8	50	<0.001
Age (years)	52.7 ± 8.1	44.9 ± 13.6**	55.8 ± 6.5	0.002
Education (years)	13.9 ± 2.4	13.7 ± 2.3	13.7 ± 3.0	NS
History of atrium fibrillation (%)	22.2	52.7		0.014
History of hypertension (%)	66.7	61.1		NS
History of diabetes (%)	14.8	3.2		NS
Previous myocardial infarction (%)	48.1	8.3		<0.001
Ejection fraction (%)	56.0 ± 7.3	62.9 ± 7.5		0.001
Cardio-pulmonary bypass time (min)	128.4 ± 35.0	128.3 ± 58.5		NS
Aortic cross-clamp time (min)	70.0 ± 21.2	76.5 ± 39.5		NS
Mini-Mental State Examination	26.2 ± 3.1	26.4 ± 2.4	27.0 ± 2.2	NS
WAIS Information subtest	24.4 ± 3.2	23.2 ± 3.6	25.8 ± 2.4	NS
WAIS Digit Span Forward	5.97 ± 1.22	6.11 ± 1.06	5.83 ± 1.27	NS
WAIS Digit Span Backward	4.30 ± 0.79	4.56 ± 1.02	4.58 ± 0.90	NS
WAIS Digit Symbol	40.0 ± 9.5	40.1 ± 10.8	45.8 ± 6.2	NS
WAIS Block Designs	34.2 ± 8.0	35.1 ± 7.8	36.9 ± 7.8	NS
Logical memory test	10.6 ± 2.3	11.0 ± 2.4	13.0 ± 2.3	0.013
Luria memory test	8.17 ± 0.61	8.09 ± 1.08	8.23 ± 0.90	NS
Benton Visual Retention Test	6.43 ± 1.48***	6.83 ± 1.50***	8.08 ± 1.44	0.007
Trail Making Test, part A	49.2 ± 6.8	51.6 ± 8.9	52.9 ± 9.8	NS
Trail Making Test, part B	86.8 ± 30.7	80.6 ± 19.3	83.1 ± 19.5	NS

Values are expressed as mean ± SD, where appropriate.

Abbreviations: CABG: coronary artery bypass grafting; NS: not significant; WAIS: Wechsler Adult Intelligence Scale.

*CABG group differed near significantly from Open Heart patients and control group.

**Open Heart patients differed significantly from CABG and control groups.

***CABG and Open Heart patients differed significantly from healthy controls.

**Table 2:** ANCOVAs of intergroup differences in change of performance at follow-up with diagnosis and sex as grouping factors and age as a covariate (Study 1).

Tests	Time	Time/Group interaction	Intergroup differences
WAIS Digit Span Forward	*F*_1,71_ = 4.27, *P* = 0.04	*F*_2,71_ = 13.7, *P* < 0.001	CABG versus Ctr; OH versus Ctr
WAIS Digit Span Backward	*F*_1,71_ = 0.12, *P* = 0.73	*F*_2,71_ = 6.03, *P* = 0.004	CABG versus Ctr; OH versus Ctr
Luria memory test	*F*_1,69_ = 0.003, *P* = 0.96	*F*_2,69_ = 3.07, *P* = 0.053	NS
Logical memory test	*F*_1,69_ = 0.07, *P* = 0.80	*F*_2,69_ = 0.60, *P* = 0.55	NS
Benton Visual Retention Test	*F*_1,69_ = 2.75, *P* = 0.10	*F*_2,69_ = 6.99, *P* = 0.002	OH versus Ctr; OH versus CABG
WAIS Block Designs	*F*_1,70_ = 7.78, *P* = 0.007	*F*_2,70_ = 18.1, *P* < 0.001	OH versus Ctr; OH versus CABG
Mini-Mental State Examination	*F*_1,71_ = 6.23, *P* = 0.015	*F*_1,71_ = 8.41, *P* = 0.001	CABG versus Ctr; OH versus Ctr
WAIS Digit Symbol	*F*_1,71_ = 3.45, *P* = 0.07	*F*_2,71_ = 24.1, *P* < 0.001	CABG versus Ctr; CABG versus OH; OH versus Ctr
Trail Making Test, part A	*F*_1,56_ = 0.01, *P* = 0.92	*F*_2,56_ = 4.20, *P* = 0.02	CABG versus Ctr; CABG versus OH; OH versus Ctr
Trail Making Test, part B	*F*_1,56_ = 0.41, *P* = 0.52	*F*_2,56_ = 4.41, *P* = 0.017	CABG versus Ctr; OH versus Ctr

Abbreviations: CABG: coronary artery bypass grafting; NS: not significant; OH: open heart surgery; WAIS: Wechsler Adult Intelligence Scale.

**Table 3:** Factor analysis of changes of non-verbal neuropsychological variables in the combined subject group.

Neuropsychological variables	Factor I	Factor II	Factor III
	Non-verbal memory	Psychomotor speed	Visuo-spatial analysis
% of variance	22.2	19.7	19.5
Benton Non-verbal Memory Test	0.798**	—	—
MMSE	0.806**	—	—
Digit Symbol Test	—	−0.678**	—
TMT A	—	0.686**	—
TMT B	—	0.289*	0.719**
Block Design test	—	—	−0.724**

*Significant Pearson's correlations between neuropsychological variables and extracted factors, *P* < 0.05.

**Correlations between neuropsychological variables and extracted factors, *P* < 0.001.

**Table 4:** Patient characteristics (Study 2).

Variables	CABG	Open heart surgery	Healthy controls	*P *
Number of patients	16	34	30	
Male sex (%)	94*	50	40	<0.001
Age (years)	56.9 ± 7.9^#^	45.2 ± 11.2	50.1 ± 15.1	0.006
Education (years)	14.1 ± 4.0	14.6 ± 2.8	14.3 ± 3.0	NS
History of hypertension (%)	75	23.5		<0.001
History of diabetes (%)	6.3	2.9		NS
Previous myocardial infarction (%)	87.5	0		<0.001
Ejection fraction (%)	59.0 ± 5.1	62.9 ± 7.3		0.050
Cardio-pulmonary bypass time (min)	121.9 ± 41.2	115.7 ± 49.6		NS
Aortic cross-clamp time (min)	69.6 ± 21.9	73.9 ± 34.6		NS
Mini-Mental State Examination	25.8 ± 3.4^#^	27.9 ± 2.4	27.3 ± 3.2	0.030
WAIS Digit Span Forward	5.76 ± 1.03	6.31 ± 1.02	6.27 ± 1.34	NS
WAIS Digit Span Backward	4.53 ± 1.07	5.11 ± 1.49	4.93 ± 1.20	NS
WAIS Digit Symbol	39.1 ± 11.5*	47.1 ± 11.8	47.6 ± 13.3	0.056
WAIS Block Designs	37.8 ± 5.5	38.9 ± 7.9	39.0 ± 8.3	NS

Values are expressed as mean ± SD, where appropriate.

Abbreviations: see Table 1.

*CABG group differed near significantly from Open Heart patients and control group.

^#^CABG group differed significantly from Open Heart patients.

**Table 5:** ANCOVAs of intergroup differences in change of performance at follow-up with diagnosis and sex as grouping factors and age as a covariate (Study 2).

Tests	Time	Time/Group interaction	Intergroup differences
WAIS Digit Span Forward	*F*_1,74_ = 0.58, *P* = 0.44	*F*_2,74_ = 15.9, *P* < 0.001	CABG versus Ctr; OH versus Ctr
WAIS Digit Span Backward	*F*_1,74_ = 0.63, *P* = 0.43	*F*_2,74_ = 0.60, *P* = 0.55	NS
WAIS Block Designs	*F*_1,74_ = 0.05, *P* = 0.83	*F*_2,74_ = 2.53, *P* = 0.087	OH versus Ctr
WAIS Digit Symbol	*F*_1,74_ = 0.18, *P* = 0.67	*F*_2,74_ = 10.3, *P* < 0.001	CABG versus Ctr; OH versus Ctr
Mini-Mental State Examination	*F*_1,68_ = 0.001, *P* = 0.98	*F*_2,68_ = 2.36, *P* = 0.10	OH versus Ctr

Abbreviations: see Table 2.
